# Biomarkers in Tumor Angiogenesis and Anti-Angiogenic Therapy

**DOI:** 10.3390/ijms12107077

**Published:** 2011-10-21

**Authors:** Andreas Pircher, Wolfgang Hilbe, Isabel Heidegger, Joachim Drevs, André Tichelli, Michael Medinger

**Affiliations:** 1 Hematology and Oncology, Innsbruck Medical University, Anichstrasse 35, 6020 Innsbruck, Austria; E-Mails: andreas.pircher@i-med.ac.at (A.P.); wolfgang.hilbe@i-med.ac.at (W.H.); 2 Department of Urology and Division of Experimental Urology, Innsbruck Medical University, Anichstrasse 35, 6020 Innsbruck, Austria; E-Mail: isabel-maria.heidegger@i-med.ac.at; 3 Tumor Center Unisantus, Custodisstrasse 3-17, 50679 Köln, Germany; E-Mail: Prof.Drevs@unisantus.de; 4 Hematology, University Hospital Basel, Petersgraben 4, 4031, Basel, Switzerland; E-Mail: tichellia@uhbs.ch

**Keywords:** angiogenesis, biomarkers, imaging techniques, vascular endothelial growth factor

## Abstract

Tumor angiogenesis has been identified to play a critical role in tumor growth and tumor progression, and is regulated by a balance of angiogenic and anti-angiogenic cytokines. Among them VEGF (vascular endothelial growth factor) and its signaling through its receptors are of crucial relevance. Inhibition of VEGF signaling by monoclonal antibodies or small molecules (kinase inhibitors) has already been successfully established for the treatment of different cancer entities and multiple new drugs are being tested in clinical trials. However not all patients are likely to respond to these therapies, but to date there are no reliable biomarkers available to predict therapy response. Many studies integrated biomarker programs in their study protocols, thus several potential biomarkers have been identified which are currently under clinical investigation in prospective randomized studies. This review intends to give an overview of the described potential biomarkers as well as different imaging techniques such as ultrasound and magnetic resonance imaging that can indicate benefit, resistance and toxicity to anti-angiogenic therapies.

## 1. Introduction

Tumor growth is crucially dependent on the development of new blood vessels, a process called angiogenesis. Since several years, tumor angiogenesis has been identified to play a critical role in tumor growth and tumor progression [1]. In adults, physiological angiogenesis is limited to a small number of specific processes, such as wound healing, tissue repair and the female reproductive cycle [2]. Pioneered by the work of Judah Folkman, it was recognized that angiogenesis plays an important role in tumor development, progression, and metastasis in solid tumors [1] but also hematological malignancies [3–5].

Tumors require nutrients and oxygen in order to grow, and new blood vessels, formed by the process of angiogenesis, provide these substrates. Tumor blood vessels are generated by various mechanisms, such as cooption of the existing vascular network, expansion of the host vascular network by budding of endothelial sprouts (sprouting angiogenesis), remodeling and expansion of vessels by the insertion of interstitial tissue columns into the lumen of pre-existing vessels (intussusceptive angiogenesis) and homing of endothelial cell precursors (EPC; CEP) from the bone marrow or peripheral blood into the endothelial lining of neovessels (vasculogenesis) [6]. Bone marrow derived progenitor cells contribute significantly to neovascularization in a variety of tumors [7,8].

The key mediator of angiogenesis is the vascular endothelial growth factor (VEGF). Therefore, VEGF and its receptors are interesting targets for anticancer therapies [9]. VEGF signaling inhibition has been shown to result in significant tumor growth delay in a wide range of animal models [10]. In the case of VEGF even a single VEGF allele knock-out led to embryonic lethality in mice [11]. The clinical benefit of this approach has also been confirmed and concentrated efforts in recent years have resulted in a number of novel anti-angiogenic agents [12]. The humanized monoclonal anti-VEGF antibody bevacizumab is the first VEGF-targeting drug, which is officially approved in patients with metastatic colorectal cancer [13], metastatic breast cancer, lung cancer, renal cell carcinomas and glioblastoma multiforme.

**Figure 1:**
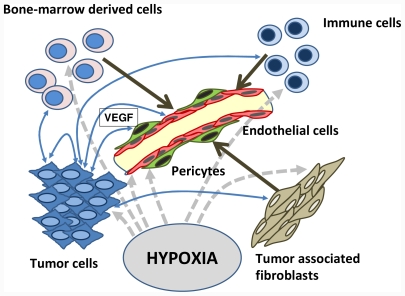
Simplified scheme of the interactions between tumor cells, bone-marrow-derived cells, and immune cells with the endothelial system. Hypoxia is a major stimulator of VEGF expression. Tumor cells produce VEGF and other pro-angiogenic factors like basic fibroblast growth factor (bFGF), platelet-derived growth factor (PDGF) and a variety of pro-inflammatory cytokines stimulating endothelial cells to proliferate. Additionally, the endothelial cells were stimulated by tumor-associated fibroblasts and bone-marrow-derived angiogenic cells (adapted from [23]. VEGF, vascular endothelial growth factor.

VEGF expression is regulated by a plethora of intrinsic and extrinsic factors. Hypoxia and hypoglycemia are major stimulators of VEGF expression [14] (Figure 1). Hypoxia-induced transcription of *VEGF* mRNA is mediated by the binding of hypoxia-inducible factor 1 (HIF-1) [15]. Cytokines may also modulate angiogenesis by regulating VEGF expression. Factors that can potentate VEGF production and this way stimulate angiogenesis include e.g. tumor necrosis factor (TNF)-α [16] and transforming growth factor (TGF)-β [17]. Intratumoral hypoxia in solid tumors has been found to be a key event in triggering angiogenesis mediated by HIF-1 and one of its downstream genes, *VEGF* [18]. Tight control of angiogenesis is maintained by a balance of endogenous anti-angiogenic and pro-angiogenic factors. VEGF has a key, rate-limiting role in promoting tumor angiogenesis and exerts its effects by binding to one of three tyrosine kinase receptors: VEGF receptor-1 (VEGFR-1), VEGFR-2 and VEGFR-3. VEGFR-1 (ligands include VEGF-A, -B and placental growth factor [PIGF]) and VEGFR-2 (ligands include VEGF-A, -C and -D) are predominantly expressed on vascular endothelial cells, and activation of VEGFR-2 appears to be both, necessary and sufficient, to mediate VEGF-dependent angiogenesis and induction of vascular permeability [9,19]. VEGF-A binds to VEGFR-1 and VEGFR-2, whereas VEGF-B as well as PlGF only binds to VEGFR-1. Both receptor tyrosine kinases are expressed in all adult endothelial cells except for endothelial cells in the brain. VEGFR-1 is also expressed on hematopoietic stem cells (HSC), vascular smooth muscle cells, monocytes, and leukemic cells [20,21]. Although the exact contribution of VEGFR-1 signaling to angiogenesis is unclear, it has been shown to co-operate directly with VEGFR-2 via heterodimerization, as well as to bind two additional VEGF homologues, VEGF-B and PIGF [22]. VEGFR-3, largely restricted to lymphatic endothelial cells, binds the VEGF homologues VEGF-C and VEGF-D and may play an important role in the regulation of lymphangiogenesis.

Further co-receptors of VEGFR are the neuropilins consisting of two genes, neuropilin-1 (NRP1) and neuropilin-2 (NRP2) [24,25]. Initially characterized as neuronal receptors, NRPs were also found to be expressed in endothelial cells and subsequently were shown to play a role in the development of the vascular system. Besides the presence of NRPs on tumor-associated vessels, NRPs were expressed by a large variety of tumors like lung cancers [26], brain tumors [27] colon cancers [28], and pancreatic cancers [29]. Targeting of both, VEGF and NRP-1 could be a more promising approach than single agent therapy [30].

Members of the FGF family are known to be angiogenic activators mediated by interactions of FGF and its receptors FGF1R and FGF2R. Several preclinical and clinical studies suggest an involvement of FGF signaling in the development of resistance to VEGF targeting agents. Recently new agents targeting the FGFR in combination with other targets are under clinical evaluation: Brivanib (BMS-582664) for example is a novel receptor tyrosine kinase inhibitor that targets the key angiogenesis receptors VEGFR-2 and FGFR [31].

Angiopoietins belong to a family of growth factors that are involved in blood vessel formation during pathological angiogenesis. The importance of Angiopoietin signaling has been recognized in transgenic mouse models as the genetic ablation of Ang-1, and its primary receptor Tie2 has led to early embryonic lethality [32]. Thus Angiopoetin inhibition represents an attractive target: AMG 386 is a promising peptide-Fc fusion protein that inhibits angiogenesis by binding angiopoietin-1 and-2 and blocking interaction with the Tie2 receptor [33].

Another target of anti-angiogenic therapies is the activin receptor-like kinase 1 (ALK1), an endothelial cell restricted receptor of the TGF-beta family. Activation of the ALK1 receptor by different ligands like bone morphogenetic protein (BMP) 9, BMP 10, and TGF-beta led to endothelial cell stimulation, proliferation and migration. For ALK1 inhibition, 3 different compounds are currently under clinical development and in phase-I evaluation [34].

Standard noninvasive imaging techniques such as ultrasonography (US), computed tomography (CT) and magnetic resonance imaging (MRI) are used to detect the success of these therapies, defined as reduction in tumor size [35]. In contrast to cytotoxic and cytostatic tumor therapies, new biological anti-cancer therapies, such as anti-angiogenic attempts, may reach biological activity at lower doses. Thus, the development of predictive biomarkers for anti-angiogenic therapies is urgently needed to select those patients most likely to derive benefit, to prevent unnecessary toxicity in resistant patients and to avoid high therapy costs.

This review focuses on the current knowledge of biomarkers in angiogenesis and its significance during anti-angiogenic therapy.

## 2. Tissue Sampling/Tissue Biomarker

Since high VEGF expression in tumors is known to correlate with advanced clinical stage and worse prognosis, VEGF expression levels in tumor tissues have been hypothesized to correlate with response and benefit from anti-angiogenic agents. Admittedly first analyses of VEGF expression levels in tissue samples of metastatic breast and colorectal cancer patients treated with bevacizumab could not verify this hypothesis [36].

Also the evaluation of microvessel density (MVD) which is known to reflect the amount of vascularization in tissue was thought to be a potential predictive marker of anti-angiogenic efficacy. Although in preclinical models MVD proved to be a predictive marker, it failed in clinical settings [37]. There are several possible explanations discussed why MVD failed to reflect antiangiogenic therapy like tumor heterogenity or intraobserveral bias.

Many studies attempted to analyze the whole VEGFR signaling cascade by different techniques and to correlate alterations with clinical response. For example, immunohistochemical (IHC) analyses of naive 21 breast cancers an increased amount of phosphorylated and thus active VEGFR2 (pVEGFR2) was observed. After therapy with bevacizumab the amount of pVEGFR2 decreased, a finding that also correlated with clinical efficacy [38]. In hepatocellular carcinoma (HCC) the treatment of sorafenib was more efficacious in patients with a highly active mitogen activated protein kinase (MAPK) determined by IHC staining of pERK [39]. The results of the validation study (SHARP trial phase III comparing best supportive care *versus* sorafenib in advanced HCC) of the MAPK activity status are awaited.

Moreover genomic analyses of the mutational status of the VEGFR (KDR) gene in angiosarcoma patients identified some mutations which correlated with increased sensitivity to VEGFR tyrosine kinase inhibitors (TKI) therapy [40]. These activating mutations occurred in 10% of the analyzed patients and correlated further with a strong KDR protein expression. Further evaluation is needed regarding EGFR (epidermal growth factor receptor), as TKIs are highly effective in patients harboring activating EGFR mutations [41].

Also germline genetic alterations in the promoter region showed to be of clinical relevance and are considered to be potential predictive markers for efficacy of anti-angiogenic therapies. The VEGF gene is highly polymorphic and many single nucleotide polymorphisms (SNPs) are described [42]. First studies from metastatic breast cancer evaluated the combination of chemotherapy plus bevacizumab compared to chemotherapy alone analyzing SNPs in the VEGF gene [43]: Different SNPs were found to correlate with a prolonged overall survival in the combination arm of chemotherapy plus bevacizumab compared with chemotherapy alone. Further SNPs were characterized and were associated with a favorable adverse event profile (less grade 3-4 hypertension). Generally hypertension is a specific side effect of anti-angiogenic therapies due to the reduced synthesis of nitric oxide (NO) in endothelial cells leading to vasoconstiction. Different studies reported an increased response rate for patients developing hypertension during anti-angiogenic therapies. Rini *et al.* [44] showed that patients with metastatic renal cell cancer (RCC) on sunitinib therapy had better response rates if they developed hypertension under sunitinib therapy. In addition a retrospective analysis of the ECOG 4599 study evaluating the addition of bevacicumab to standard chemotherapy in NSCLC (non-small cell lung cancer) observed that elevated blood pressure levels during bevacizumab therapy was associated with improved therapy outcomes [45]. Recently Maitland *et al.* published recommendations of a cardiovascular toxicity expert panel for an optimized treatment of hypertension induced by anti-angiogenic therapies [46].

Another important point is that tissue samples allow to evaluate the stromal compartment and the tumor microenviroment. Many preclinical studies showed that stromal interactions like myeloid cells and fibroblast interactions lead to acquired resistance to anti-angiogenic agents [47]. Cascone *et al.* [48] showed in a mouse model that during anti-angiogenic therapy in the stromal compartment EGFR and FGFR (fibroblast growth factor receptor) mediated pathways were upregulated and inhibited efficacy of anti-angiogenic agents. Thereafter combined inhibition of EGFR and anti- VEGF restored the antitumor effect.

Also tumor infiltrating myeloid cells showed an impact on efficacy of anti-angiogenic therapies. In various preclinical studies myeloid cells caused a stimulation of the angiogenic network independently of the VEGF signaling cascade consequently contributing to an acquired resistance against antiangiogenic therapies [49]. This subpopulation of myeloid cells (CD11b+Gr1+ cells) can be mobilized by G-CSF, IL-6 and SDF1α from the bone marrow [50]. CD11b+Gr1+ cells were further characterized by the expression of the Bv8 protein [51]. Bv8 is related to the endocrine gland derived VEGF and was first extracted from the skin of a frog [52]. Functional analyses of Bv8 showed to induce mobilization of myeloid cells from the bone marrow and to circumvent VEGF mediated angiogenesis [53]. Therefore Bv8 is an attractive target and first inhibition studies of Bv8 in preclinical models with neutralizing antibodies proved tumor shrinkage and angiogenesis inhibition [54].

In conclusion, *in situ* biomarker evaluation of predictive biomarkers is an important tool for translational research studies, but limited by the invasiveness of tissue sampling and also by the heterogeneity of the tumor tissue.

## 3. Blood Soluble Markers

The evaluation of angiogenic parameters in serum/plasma samples with standard immunogenic assays is another attractive method for monitoring anti-angiogenic therapies not only because of its feasibility and its low costs.

However in four different phase III studies evaluating bevacizumab in combination with chemotherapy (AVF2107g, E4599, AVAiL and AVOREN) baseline VEGF levels were not able to predict response to anti-angiogenic therapy [55]. In contrast to these findings, Dowlati *et al.* showed that high VEGF levels at baseline correlated with high response rates to bevacizumab therapy (in combination with standard chemotherapy) [56].

Briefly, these early biomarker studies showed that angiogenesis is a complex network regulated by a variety of cytokines and that the determination of a dynamic marker profile including more than one single candidate biomarker could be of relevance.

Subsequent studies evaluated beside VEGF numerous circulating angiogenic factors (CAFs, up to 40 angiogenic and immunological markers at defined timepoints). In metastatic colorectal cancer Kopetz *et al.* [57] analyzed a CAF panel during FOLFIRI plus bevacizumab therapy and could demonstrate that several measured cytokines like bFGF, PlGF, MMP-9 and HGF or PDGF were altered during therapy. The authors observed increased CAFs like before disease progression occurred, so that CAF levels of the blood stream predicted resistance to anti-angiogenic therapies.

Preclinical models hypothesized that during VEGF blockade other important pro-angiogenic cytokines are upregulated, thus the anti-angiogenic effect could be restored by blockade of the upregulated factors. In addition, a subset of factors associated with myeloid cell recruitment (monocytes) was elevated at disease progression underlying the hypothesis that immune cells contribute to evasive resistance mechanisms against anti-angiogenic agents. In a similar study NSCLC (non-small cell lung cancer) patients were treated with the VEGFR TKI vadentanib monotherapy or with a combination of chemotherapy plus vandetanib [58]. At four different timepoints a panel of 35 CAFs was evaluated showing an increase of IL8 and VEGF during vandetanib therapy and a concurrent decrease of sVEGFR2. This observed increase of VEGF2 and its corresponding decrease of sVEGFR2 might be a specific effect of anti-angiogenic therapies, a finding which could be due to increased tumor hypoxia causing an increased VEGF production.

This inverse correlation was observed already in many preclinical models and the first clinical observation was described by Norden-Zfoni *et al.* 2007 in gastrointestinal stromal tumors treated with sunitinib [59]. In summary, currently VEGF and sVEGF2R determination represents one of the best available options for predicting anti-angiogenic efficacy.

Another small study evaluated CAF signature, which included HGF and IL-12 in neoadjuvant treated NSCLC patients with pazopanib [60]. They found that CAFs were associated with response to pazopanib and identified responding patients with 81% accuracy. This was the first study reporting that increased levels of IL-12 (master regulator of TH1 immune response) are good predictors for response to anti-angiogenic therapies.

In summary, to date the relevance of soluble biomarkers in the blood is not entirely investigated due to the fact that most candidate biomarkers were evaluated retrospectively and prospective validation is missing.

## 4. EPC

Circulating endothelial cells (CEC) are generally known to be increased in cancer patients. CECs are endothelial cells originating from blood vessel walls expressing the phenotype of mature endothelial cells. Elevated CEC counts have been associated with several diseases including myocardial infarction and transplant reactions. Moreover there is evidence that CECs are increased in cancer patients [61,62].

Recent studies showed that CEC counts vary during anti-angiogenic therapy: In a breast cancer model it has been shown that CECs positively correlated with tumor invasiveness and tumor volume [63]. Thus it is speculated that CECs mirror a vascular remodeling due to high VEGF levels produced by cancer cells. Therefore CECs possibly can be used as marker for angiogenesis and consequently for monitoring anti-angiogenic therapies.

Different clinical trials tent to measure CEC levels during anti-angiogenic therapy and a positive correlation between decreased CEC numbers and progression-free survival (PFS) has been observed [64,65]. Moreover greater baseline levels of CECs have been correlated with therapy response to anti-angiogenic therapy; however at the time of progression there was an observable decrease in CEC amount [66]. Interestingly this CEC decrease at time of disease progression resulted in increased VEGF and FGF levels.

Taken together, measuring CEC level before anti-angiogenic therapy, could be a predictive biomarker for therapy response. Moreover CEC measurements during anti-angiogenic therapy are possibly able to anticipate resistance of anti-angiogenic agents.

Similar to CEC also circulating progenitor cells (CEP) are elevated in cancer patients associated with disease progression [67]. In contrast, CEPs are a cell population originating from the bone marrow expressing stem cell defining markers. Mechanistically there are several hints that CEPs stimulate vascularization triggered by VEGF levels, mobilizing CEPs from the bone marrow so that CEPs are key contributors of tumor vascularization. Several studies found elevated CEP counts in several cancer entities including lung, breast and brain cancer; in some of these studies high CEP levels were found to correlate with poor prognosis and consequently with poor survival rates. Moreover CEPs were found in preclinical and clinical studies to play a possible role in the development of resistance mechanisms against anti-angiogenic therapy and to chemotherapeutic agents [68]. Thus there is intensive research ongoing to define therapeutic strategies to reduce CEP mobilization thereby enhancing on one hand the efficacy of anticancer therapeutics and on the other hand to reduce the risk of developing metastases.

To summarize, although several studies observed relationships between CEP and tumor neo-angiogenesis these findings are still a matter of debate due to the fact that there is no standardly defined immunophenotyp and functional characterization of this cell population. Furthermore CEC and CEP in peripheral blood is rarely measurable and therefore reproducibility and standardization of techniques is difficult.

## 5. Imaging Techniques

### 5.1. DCE-MRI

In the absence of predictive biomarkers for antiangiogenic strategies, various imaging techniques are being examined as potential pharmacodynamic markers, including dynamic contrast-enhanced magnetic resonance imaging (DCE-MRI). Several clinical trials describe a decrease in tumor perfusion in response to anti-angiogenic treatment [35]. DCE-MRI is a non-invasive functional imaging technique that permits indirect measurement of tumor hemodynamics [69,70]. It may therefore be suitable for monitoring the effects of VEGF signaling inhibitors on tumor vasculature. DCE-MRI, using gadolinium chelate as the contrast agent, is a useful technique to study the pathophysiology of tumors, particularly with respect to vascular perfusion and permeability. The MRI enhancement is due to increasing gadolinium concentration, largely in the extravascular and extracellular space and depends on tumor perfusion, tumor vascularity and tumor vascular permeability. Gadolinium diffuses passively from the blood vessel into the extracellular space at a rate proportional to the concentration difference. Once in the extracellular space it disperses freely without binding and will efflux back into the vascular space in a similar manner with a similar rate constant. Ki or K^trans^ is an objective measurement quantifying the rate constant for this process.

DCE-MRI utilizes a low molecular weight paramagnetic contrast agent such as gadolinium-DTPA, which readily diffuses from the blood to the extravascular extracellular space. By acquiring a set of rapid MR images, the time course of the change in T1 relaxation time induced by the contrast agent may be followed. Contrast agent concentration can be calculated from T1 relaxation times using the known linear relationship [69]. The time course obtained can be characterized by the initial area under the contrast agent concentration-time curve (iAUC) or a pharmacokinetic model may be applied. With the latter, the data are fitted to estimate the transfer of contrast agent between the plasma and the extracellular, extravascular space (the transfer constant K^trans^). Although iAUC and K^trans^ are incompletely validated endpoints that are sensitive to changes in a number of hemodynamic parameters, including blood flow, blood volume, vessel permeability and vessel surface area [71], emerging data from several early-phase clinical trials of VEGF signaling inhibitors have shown changes in K^trans^ and/or iAUC that are consistent with reductions in VEGF-dependent tumor perfusion and vascular permeability [72–74].

The method was first described preclinically using vatalanib (formerly PTK787/ZK 222584), a specific inhibitor of the VEGF–receptor tyrosine kinases, which showed anti-tumor and anti-angiogenic activity in a murine renal cell carcinoma (RENCA) model. In this model, after intrarenal application of RENCA cells, mice developed a primary tumor and metastases to the lung and abdominal lymph nodes [35,75,76]. After daily oral therapy with either vatalanib or vehicle, primary tumors of all animals were analyzed by DCE-MRI. Gadolinium-DOTA was used as the contrast agent for detecting vessel permeability and contrast agent extravasation. Vatalanib treatment led to a significant decrease in vessel permeability. Furthermore, increase in partial blood volume was found in the vatalanib-treated group, although vessel density was reduced as seen by histology [75]. Using the corrosion cast technique, reduction in vessel density was significant but not very pronounced, predominantly due to the loss of microvessels.

In the clinical phase-I study, patients with colorectal cancer were treated with oral, once-daily vatalanib at doses ranging from 50 to 2000 mg day [69]. In that study, early changes in tumor vascularity and vascular permeability (Ki) were assessed by DCE-MRI as biomarkers of clinical activity and correlated with vatalanib pharmacokinetics and subsequent clinical outcome after 56 days of treatment. A 60% decrease in Ki was significantly correlated with non-progressive disease after two cycles of vatalanib treatment.

In another phase-I study with cediranib (AZD2171, Recentin®), a potent inhibitor of both VEGFR-1 and VEGFR-2, DCE-MRI was used to evaluate the anti-angiogenic activity of cediranib [74]. Patients with solid tumors and liver metastases refractory to standard therapies received once daily oral AZD2171 (0.5 to 60 mg). Altogether eighty-three patients received cediranib. Pharmacodynamic assessments demonstrated time-, dose-, and exposure-related decreases in initial area under the curve, defined over 60 seconds post-contrast arrival in the tissue (iAUC_60_) using dynamic contrast-enhanced magnetic resonance imaging, as well as dose- and time-dependent reductions in soluble VEGF receptor 2 levels. The reduction in iAUC_60_ on day 2 observed in this study suggests a reduction in vascular permeability, whereas the sustained effects on days 28 and 56 are likely to reflect changes in blood flow. Overall, the findings from the DCE-MRI investigation demonstrated that cediranib modulates tumor vascular physiology and reduces tumor blood flow and vascular permeability [74].

DCE-MRI is a promising biomarker for assessing anti-angiogenic treatment. However, standardized methods are required to establish reliable sources in evaluation of antitumor therapy. In contrast to previous methods, in which MRI was added as a clinical endpoint, prospective studies are needed to test standardized DCE-MRI methods and to establish MRI as a biomarker itself, which can be used in very early stages of drug development, to monitor anti-tumor effects and correlate them with existing prospective biomarker performance. Quantification by use of pharmacokinetic modeling necessitates calibration to determine the relationship between concentration of the contrast agent and signal intensity; however, there is no simple relationship between contrast concentration and signal-intensity, especially at high contrast concentration [77].

### 5.2. Ultrasound

Ultrasound is a favored modality for imaging angiogenesis because it does not expose patients to any radiation risks, it is widely available, can be repeated often and it is mobile. However, B-Mode ultrasound remains limited in imaging angiogenesis because small vessels cannot be detected [35]. Knowledge about ultrasound and especially Doppler ultrasound has been dramatically improved in recent years, mainly by software developments. Color Doppler reveals the vascular anatomy of tumors—even the branching pattern, shunts and blind ending vessels can be visualized—and are standardized with visual scoring systems. Early data produced in animal studies indicated that results from Doppler methods are not correlated to the absolute micro-vessel density because they can visualize only larger vessels but the measurement of blood flow velocity with color Doppler ultrasound can be correlated with effects from anti-angiogenic therapy.

In a study addressing animal models like murine renal cell carcinoma (RENCA), developing a primary tumor and metastases to the lung and abdominal lymph nodes after intrarenal application of tumor cell color Doppler imaging ultrasound was evaluated [76]. Primary tumors were located in all animals using a sonograph having a direct-contact high frequency ultrasound transducer. The left renal artery was detected by color Doppler imaging, and measurements of systolic and diastolic blood flow velocity and resistance index were performed and compared between animals that were treated with an anti-angiogenic agent or not. Taking the values from the control vessel (abdominal aorta) into account, reduction of systolic blood flow velocity in animals treated with an anti-angiogenic agent could be confirmed, showing a reduction in systolic blood flow velocity by 44% [76].

In the same RENCA model, ZD6126, a vascular-disrupting agent that affects the endothelial tubulin cytoskeleton causing selective occlusion of tumor vasculature and extensive tumor cell necrosis, was examined. ZD6126 treatment led to a significant reduction in tumor size and was associated with extensive tumor necrosis and a reduction in tumor blood flow measured by Doppler imaging ultrasound technique [78].

In a clinical study with vatalanib in patients with colorectal cancer treated with oral, once-daily vatalanib was designed to evaluate contrast-enhanced color Doppler imaging as a biomarker for anti-angiogenic treatment [79]. As inclusion criteria, a tumor vessel in a liver metastasis had to be present with a good signal-to-noise ratio. Blood flow measurements were performed at baseline, day 3 and at day 28 as well as after each additional treatment cycle. The systolic and diastolic blood flow was measured in the tumor vessel as well as in the hepatic artery. Heart rate and blood pressure measurements were measured. The resistance index (RI) was calculated as follows: RI = (*V*_sys_ – *V*_dia_)/*V*_sys_. In contrast to the animal studies, no significant dose-related changes could be described for the RI. There was a trend to a higher blood flow with increasing doses of vatalanib but the results are not significant due to the very small sample size [79].

The future role of ultrasound as well as contrast-enhanced color Doppler imaging in anti-angiogenic agents will be defined by addressing the challenges of examiner-dependent reproducibility of results.

## 6. Biomarkers under Anti-Angiogenic Therapies

### 6.1. Anti-Angiogenic Therapies

**Figure 2:**
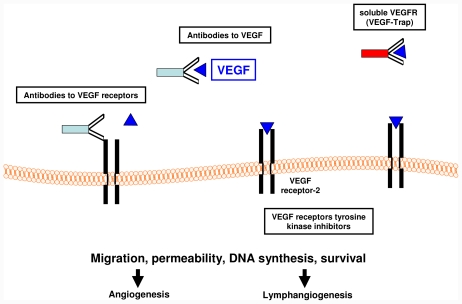
Therapeutic strategies to target the VEGF/VEGF receptor system (adapted from [5]). VEGF, vascular endothelial growth factor.

Anti-angiogenic therapies are mostly based on inhibiting the binding of VEGF to VEGFR by neutralizing antibodies to the ligand or to the receptor, soluble receptors, small molecule inhibitors or are directed against the tyrosine kinase activity of the VEGF receptors [5] (Figure 2). We will focus on several molecules interfering with the VEGF/VEGFR system, which already have been approved or are currently evaluated in clinical trials for solid tumors.

### 6.2. Receptor Tyrosine Kinase Inhibitors

Small molecule VEGF receptor tyrosine kinase inhibitors are a further important class of anti-angiogenic drugs.

### 6.3. Sorafenib

Sorafenib (Nexavar^®^) is a multikinase inhibitor of VEGFR2-3, PDGFR, Raf kinase and c-Kit the receptor of stem cell factor, currently approved for the treatment of advanced HCC and renal cell carcinoma (RCC) [5,80]. In a phase-II trial evaluating sorafenib in previously treated advanced NSCLC assessed possible predictive biomarkers in 34 evaluable patients [81]. Among them the effect of VEGF increase and sVEGFR2 decrease was observed again, further decreased bFGF levels were correlated with increased survival and disease progression. Also functional imaging with DCE-MRI was performed and *K_ep_* showed a significant predictive value for overall and progression-free survival.

Hypertension is known to be a class specific side effect of anti-angiogenic agents due to inhibited NO production in endothelial cells. Maitland and colleagues found that sorafenib administration leads to a blood pressure raise within 24 hours [82].

### 6.4. Cediranib

Cediranib (AZD2171; Recentin^®^) is a potent inhibitor of both VEGFR-1 and VEGFR-2; it also has activity against c-kit, PDGFR-β, and FLT4 at nanomolar concentrations [83]. Cediranib has been shown to inhibit VEGF signaling. In a phase-I study, cediranib was well tolerated up to 45mg/d in patients with a broad range of solid tumors [74]. The most common toxicities include diarrhea, dysphonia, and hypertension. Pharmacodynamic assessments demonstrated time-, dose-, and exposurerelated decreases in initial area under the curve, defined over 60 seconds post-contrast arrival in the tissue (iAUC60) using DCE-MRI, as well as dose- and time-dependent reductions in soluble VEGF receptor 2 levels [74]. In a phase-I study with cediranib in 35 patients with acute myeloid leukemia (AML), the most common adverse events were diarrhea, hypertension and fatigue. Dose and time-dependent reductions in sVEGFR-2 were observed, and there was a positive correlation between cediranib exposure and the change in plasma VEGF levels from baseline [84]. Blood pressure elevation seems to be a class-specific side effect of most anti-angiogenic agents (sorafenib, sunitinib, bevacizumab and axitinib) due to the decreased production of nitric oxide and prostacyclines in vascular endothelial cells. The hypertension can be treated with standard medication. Therefore, blood pressure monitoring during anti-angiogenic therapy is recommended [85].

In a phase-II study randomized, factorial, double-blind study of cediranib in patients with advanced solid tumors, the hypertension management was prospectively investigated [85]. All patients developing hypertension on cediranib treatment were treated with a standardized, predefined hypertension management protocol. Antihypertensive prophylaxis did not result in fewer dose reductions or interruptions. Increases in blood pressure, including moderate and severe readings of hypertension, were seen early in treatment in all groups and successfully managed. Severe hypertension occurred in one patient receiving prophylaxis *versus* 18 in the nonprophylaxis groups. They concluded that early recognition and treatment of hypertension is likely to reduce the number of severe hypertension events. This protocol is included in all ongoing cediranib clinical studies.

### 6.5. Sunitinib

Sunitinib (Sutent^®^) is a multikinase inhibitor of VEGFR 1–3, RET and PDGFRα/β, currently approved for the treatment of RCC, imatinib-resistant gastrointestinal stromal tumors (GIST) and pancreatic neuroendocrine tumors (pNET) [86–88].

Many studies on sunitinib included biomarker programs to identify predictive markers for response. Perez-Gracia *et al.* anyalzed a large panel of 174 cytokines before and after treatment with sunitinib and found that baseline TNF-alfa and MMP-9 levels were predictive for sunitinib activity [89]. In contrast, Farace *et al.* found no correlation between CAFs and response to sunitinib therapy, but they described an increase of CEP as marker for therapy response [90]. Another study evaluated a panel of 16 biomarkers involved in tumor pathways targeted by sunitinib, using real-time quantitative reversetranscriptase PCR in patients with metastatic RCC [91]. Only the levels of VEGF soluble isoforms (VEGF_121_ and VEGF_165_) were associated with response to sunitinib therapy and the ratio of VEGF_121_/VEGF_165_ was a strong prognostic marker.

Biomarker analyses of HCC patients treated with sunitinib showed a correlation between decreased levels of IL-6 and soluble c-KIT and a delayed tumor progression; vice versa elevated levels of IL-6 and c-KIT were associated with an unfavorable disease course [92]. In this study no correlation of VEGF cytokines (like VEGF, sVEGFR) and therapy response was observed. However, responders to sunitinib therapy could be detected by a decrease of the vascular permeability measured by DCE-MRI, which was correlated with a delayed progression.

### 6.6. Anti-VEGF Monoclonal Antibodies

#### Bevacizumab

Bevacizumab (Avastin^®^) is a humanized monoclonal antibody IgG1 which was created from a murine anti-human VEGF monoclonal antibody that blocks the binding of human VEGF to its receptors, thereby disrupting autocrine and paracrine survival mechanisms mediated by VEGFR-1 and VEGFR-2 [93]. Bevacizumab is the first VEGF targeting drug, which has been officially approved for cancer therapy. Bevacizumab demonstrated survival benefits in patients with metastatic colon cancer when combined with conventional chemotherapy [13]. Since then, it has been tested in several other cancer types.

The determination of the optimal biological dose of bevacizumab for clinical use was challenging and not conclusive, as shown by the various doses of bevacizumab used in phase III trials [94,95]. Inhibition of VEGF signaling may induce a hypertensive response. Bevacizumab inhibits VEGF signaling to endothelial cells, which leads to rapid rises in blood pressure [96]. Hypertension, therefore, could be a useful surrogate marker of VEGF activity and predict the anti-angiogenic activity of bevacizumab [96].

In a recent study, hypertension was examined as biomarker in patients with metastatic colorectal cancer treated with chemotherapy and bevacizumab [97]. Overall, 57 patients (56%) developed ≥ grade 1 hypertension (median blood pressure 168/97 mm Hg), whereas 44 (44%) remained normotensive when treated with bevacizumab-containing chemotherapy regimen. Overall response rate was higher among patients with hypertension (30 *vs.* 20%; *p* = 0.025). Hypertension was associated with improved progression-free survival (10.5 *vs.* 5.3 months; *p* = 0.008) and overall survival (25.8 *vs.* 11.7 months; *p* < 0.001) and development of hypertension within 3 months had an independent, prognostic influence in a multivariate landmark survival analysis together with other known colorectal cancer prognostic factors (*p* = 0.007).

In a phase-III study of bevacizumab in combination with interferon alfa *versus* interferon alfa montherapy in patients with metastastic renal cell carcinoma, patients in the combination arm had significantly more grade 3 to 4 hypertension [98]. Patients who developed hypertension on bevacizumab plus interferon alfa had a significantly improved progression-free survival and overall survival *versus* patients without hypertension. Contrary, an analysis of 5,900 patients across 6 phase-III studies of metastatic cancer treated with bevacizumab was performed by Hurwitz *et al.* [99]. In 5 of 6 studies, hypertension during bevacizumab treatment was not predictive of clinical benefit or prognostic for the course of disease.

Soluble plasma and serum markers of angiogenesis and of activated endothelial cells can also be used to assess anti-angiogenic activity [100]. VEGF levels can be predictive of responding to anti-angiogenic therapy [79,80].

In a recent study, molecular and genetic markers to predict or monitor the efficacy of bevacizumab was investigated in patients of metastatic colorectal cancer [101]. Plasma levels of VEGF, PlGF, sVEGFR-2 and thrombospondin-1 were assessed by ELISA assay at different time points in a cohort of 25 patients enrolled in a phase-II trial. Treatment with FOLFOXIRI plus bevacizumab determined a prolonged and significant reduction in plasma free, biologically active VEGF concentration. Interestingly, VEGF concentrations remained lower than at baseline also at the time of progressive disease.

In a phase-II/phase-III trial, patients with advanced NSCLC were randomized to carboplatin + paclitaxel (PC arm) or PC + bevacizumab (BPC arm) [56]. VEGF, basic fibroblast growth factor (bFGF), soluble intercellular adhesion molecule (ICAM) and E-selectin was measured pretreatment and during therapy. Baseline ICAM showed significant associations with response and survival in both groups. Patients with low baseline ICAM had a higher response rate (32% *vs.* 14%; p=0.02), better overall survival (*p* = 0.00005), and better 1-year survival (65% *vs.* 25%) than those with high ICAM, respectively, regardless of treatment arm. Patients with high VEGF levels were more likely to respond to BPC compared with PC, but this was not predictive of survival. The results also suggest a benefit from bevacizumab for patients with low baseline ICAM levels (53% reduction in the progression-free survival hazard rate).

In a recent study, predictive biomarkers were investigated in patients with metastatic breast cancer treated with bevacizumab and capecitabine [55]. Biomarker expression was assessed by *in situ* hybridization (VEGF-A, VEGF-B, thrombospondin-2 and Flt4) or immunohistochemistry (VEGF-C, PDGF-C, neuropilin-1, delta-like ligand (Dll) 4, Bv8, p53 and thymidine phosphorylase) on formalin-fixed, paraffin-embedded tissue. Patients with low scores for Dll4, VEGF-C, and neuropilin-1 showed trends toward improvement in progression-free survival associated with the addition of bevacizumab to capecitabine.

Regarding DCE-MRI to measure non-invasive efficacy of bevacizumab therapy in cancer patients, a phase-II trial examined the additional biomarker effect on angiogenesis when bevacizumab is added to docetaxel in patients with inoperable breast cancer [102]. Plasma and serum markers of endothelial damage, DCE-MRI, and tumor microvessel density were assessed before treatment and at the end of each cycle. VEGF increased during treatment; more so with docetaxel-bevacizumab than docetaxel alone (*p* < 0.0001). DCE-MRI showed a greater decrease in tumor perfusion calculated by initial area under the curve for the first 90 seconds in docetaxel-bevacizumab than docetaxel alone (*p* = 0.024). DCE-MRI also showed an overall decrease in tumor volume (*p* = 0.012).

## 7. Conclusions

Angiogenesis is essential in the development of malignancies. In such instances, VEGF/VEGFRrelated pathways are the most relevant regulators of neoangiogenesis, vasculogenesis, and recruitment of endothelial progenitor cells. Furthermore, VEGF/VEGFR interactions can stimulate proliferation, migration, and survival of tumor cells. Anti-angiogenic therapies are integrated in the treatment management of many different tumor entities. However, not all patients respond to therapy and only a few benefit by progression-free survival. Thus, it is important to find predictive biological markers of objective response, as the response rate correlates with overall survival, or involved in resistance to anti-angiogenic drugs in order to improve therapy efficacy, or to propose alternative anti-angiogenic therapy in case of treatment failure. Most of the promising soluble, tissue and imaging biomarkers will need to be validated in prospective trials as currently no “ideal” biomarkers exist which identifies patients most likely to receive the greatest benefit from anti-angiogenic therapy.
